# Methylation of *p16* ^*ink4a*^ promoter is independent of human papillomavirus DNA physical state: a comparison between cervical pre-neoplastic and neoplastic samples

**DOI:** 10.1590/0074-02760180456

**Published:** 2018-12-17

**Authors:** Fernanda Nahoum Carestiato, Sergio Menezes Amaro-Filho, Miguel Angelo Martins Moreira, Silvia Maria Baeta Cavalcanti

**Affiliations:** 1 Universidade Federal Fluminense, Departamento de Microbiologia e Parasitologia, Niterói, RJ, Brasil; 2 Instituto Nacional de Câncer, Programa de Genética, Rio de Janeiro, RJ, Brasil

**Keywords:** HPV16, cervical cancer, *p16*^*ink4a*^, methylation, integration

## Abstract

BACKGROUND Epigenetic modifications in host cells, like *p16* ^*ink4a*^ methylation, have been considered as putative complementary mechanisms for cancer development. Because only a small proportion of infected women develop cervical cancer, other factors might be involved in carcinogenesis, either independently or in association with high-risk human papillomavirus (HR-HPV) infections, including epigenetic factors. OBJECTIVES We hypothesised that *p16* ^*ink4a*^ methylation might have a role in cancer development driven by HPV16, mainly in the presence of intact *E1/E2* genes. Thus, our objectives were to assess the status of *p16* ^*ink4a*^ methylation and the HPV16 *E1/E2* integrity in samples in different stages of cervical diseases. METHODS Presence of HPV16 was determined by E6 type-specific polymerase chain reaction (PCR). Methylation status of the *p16* ^*ink4a*^ promoter was assessed by methylation-specific PCR in 87 cervical specimens comprising 29 low-grade (LSIL), 41 high-grade (HSIL) lesions, and 17 cervical cancers (CC). Characterisation of E1 and E2 disruption (as an indirect indicator of the presence of episomal viral DNA) was performed by PCR amplifications. FINDINGS We observed a significantly increased trend (*nptrend* = 0.0320) in the proportion of methylated *p16* ^*ink4a*^ in cervical samples during cancer development. Concomitant *E1* and *E2* disruptions were the most frequent pattern found in all groups: CC (76%), HSIL (54%), and LSIL (73%). No statistically significant differences between *p16* ^*ink4a*^ methylation and *E1/E2* integrity, in histological groups, was observed. MAIN CONCLUSIONS There was an increase in methylation of the *p16* ^*ink4a*^ promoter from pre-neoplastic lesions to cancer. Additionally, a high frequency of *E1/E2* disruptions in LSIL/HSIL suggested that viral DNA integration was an early event in cervical disease. Moreover, the methylation status was apparently independent of HPV16 integrity.

The International Agency for Research on Cancer (IARC) recognises 12 types of high-risk (HR) oncogenic human papillomavirus (HPVs) (HR-HPV16, 18, 31, 33, 35, 39, 45, 51, 52, 56, 58, and 59) due to their high prevalence (> 99%) in cervical cancer (CC) and profuse molecular evidence.^1^ HPV16 is the most prevalent genotype worldwide, either in cytologically normal samples (20.4%), pre-neoplastic lesions (25.1-47.5%) or cervical tumors (62.6%).^2^

HR-HPV induced oncogenesis is strongly associated with overexpression of E6 and E7 viral oncoproteins and inhibition of the most relevant tumor-suppressor pathways, represented by p53 and pRB cell proteins.^3^ Generally, HR-HPV-E6 forms a protein complex with p53 followed by proteasome mediated degradation, thus affecting the p53-induced pathway of growth-arrest and apoptosis.^3^ On the other hand, it also interferes with the retinoblastoma gene protein (pRB) that controls the expression of proteins required for cell-cycle progression following association with the E2F transcription factor.^3^ In non-infected hosts, phosphorylation of pRB induced by cyclin D: cyclin dependent kinases 4 and 6 (cyclin D:CdK4/6) releases E2F, driving the expression of proteins involved in the progression of G1 to S-phase.^3^ By a negative feedback mechanism, P16 level rises and regulates the level of cyclin D:CdK4/6, inhibiting pRB phosphorylation, thus allowing for formation of pRB/E2F complexes that eventually block cell cycle progression.^3^ In the presence of HR-HPV, the E7 oncoprotein binds to pRB, maintaining a high level of free E2F, activating proliferation, independent of cyclinD/CdK4/6 induced phosphorylation.^3^ ^,^ ^4^ Consequently, p16 accumulates in cells, in the absence of a feedback-mediated regulation, a reason for the association of *p16* ^*ink4a*^ overexpression in cervical specimens with HR-HPV transformation.^3^ ^,^ ^5^ ^,^ ^6^

HR-HPV E6 and E7, moreover, generate severe chromosomal instability, favouring HPV DNA disruption and integration into the host genome. This leads to increased expression and stability of E6 and E7 transcripts due to disruption of the viral genome, which frequently occurs upstream or at the *E2* gene, resulting in E2 inactivation and cancer development. This is because *E2* encodes a dose-dependent transcriptional repressor of the *E6/E7* HPV DNA promoter at the 3’LCR.^7^ ^,^ ^8^ ^,^ ^9^ However some studies have shown that approximately 40% of cervical tumors harbour episomal or concomitant forms of HPV16 DNA, indicating the presence of a functional E2 protein.^10^ ^,^ ^11^ ^,^ ^12^

Furthermore, since only a small proportion of infected women develop cervical cancer, other factors might be involved in carcinogenesis, either by themselves or in association with HR-HPV infections, including environmental, immunologic, behavioral, hormonal, genetic and epigenetic factors.^13^ ^,^ ^14^ Among genetic and epigenetic factors, inactivation of the *p16* ^*ink4a*^ gene (cyclin-dependent kinase inhibitor 2a, CDKN2A) by point mutations, deletions, and methylation, has been reported in a large number of other cancers, an event that might also contribute to cervical cancer development.^15^

We hypothesised that cases with intact *E1/E2* genes, suggestive of an HPV episomal conformation, might be associated with a higher methylation pattern at the *p16* ^*ink4a*^ promoter than cases with disrupted genes, thus contributing to cancer development, mainly in early events. In this paper, we assessed the methylation status of the *p16* ^*ink4a*^ promoter and the integrity of *E1/E2* of HPV16 DNA in pre-neoplastic and neoplastic cervical samples. An increased methylation trend of the *p16* ^*ink4a*^ promoter, from pre-neoplastic to cervical cancer, was found in HPV16+ patients, with a high frequency of *E1/E2* disruptions in low-grade squamous intraepithelial cells/high grade squamous intraepithelial cells (LSIL/HSIL) samples. However, the methylation status was apparently independent of HPV16 integrity.

## MATERIALS AND METHODS

*Samples and study design* - All patients filled out a questionnaire and signed a written consent. Cervical samples were collected with brushes in a double-blind protocol from patients attending Moncorvo Filho Hospital, Rio de Janeiro, Brazil, between 2005 and 2014.

**Fig. 1:**
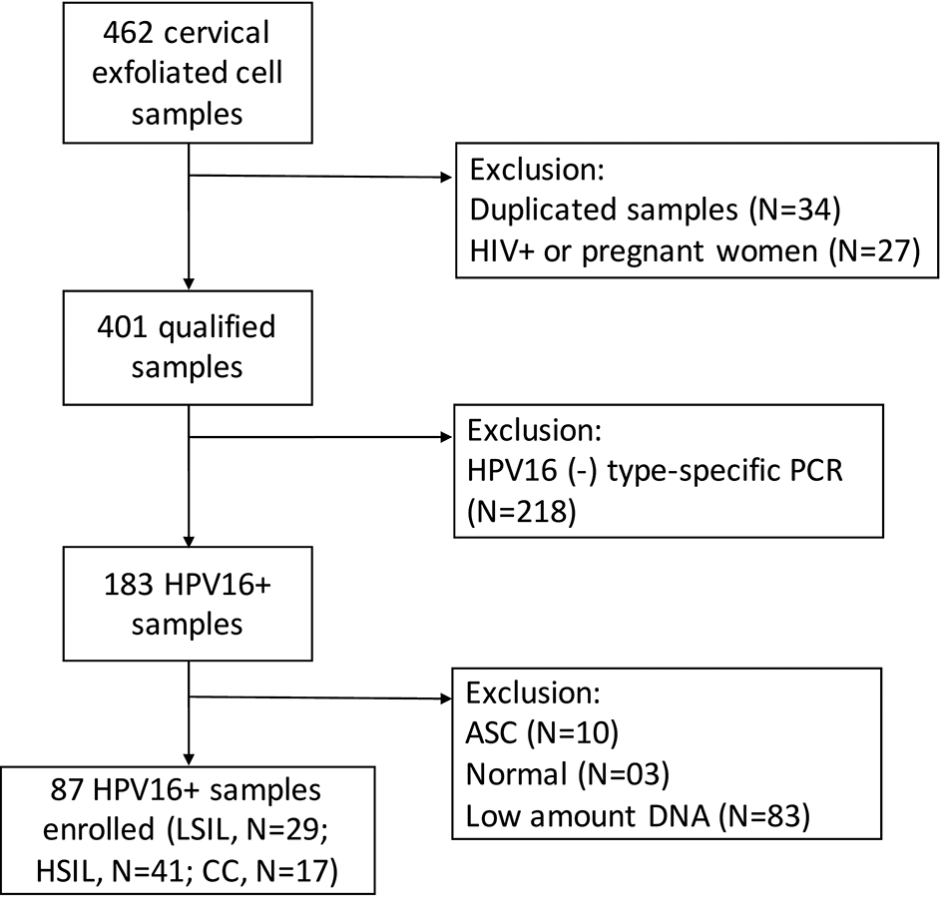
flowchart displaying sample distribution from initial population to total samples herein studied. Low amount of DNA was measured, depicted by a weak amplification band from type-specific polymerase chain reaction (PCR). ASC = atypical squamous cells; LSIL = low-grade squamous intraepithelial cells; HSIL = high-grade squamous intraepithelial cells; CC = cervical cancer.

An initial set of 462 exfoliated cell samples, displaying a broad spectrum of cervical pathology, from low-grade lesions to high-grade lesions and cancer, was collected. Samples were collected for standard cytopathology according to the Bethesda System and for molecular analysis in Tris-EDTA-SDS (10 mM Tris-HCl, pH 7.5; 1 mM EDTA; 0.6% SDS) buffered solution and frozen at -20ºC. Sixty-one samples were excluded from analysis, 34 due to duplications and 27 from HIV infected or pregnant women. Of the 401 remaining samples, 183 were HPV16+, accounting for a prevalence of 46% in this cohort. Ten patients with normal cytology and three others with atypical squamous cells (ASC) were further excluded due to the small size of this sample set. Moreover, samples with apparently low HPV concentration, showing weak amplification following type-specific polymerase chain reaction (PCR), were also excluded because a higher amount of DNA was required for evaluating the physical conformation of the viral genome (see below). Finally, 87 HPV16+ patients were enrolled in this study, encompassing 29 cases of LSIL, 41 HSIL and 17 CC. A flowchart displaying sample selection is summarised in Fig. 1.

*DNA isolation, HPV DNA detection and typing* - Cervical samples were incubated at 56ºC for 2 h in digestion buffer (10 mM Tris-HCl, pH 7.5; 1 mM EDTA; 0.6% SDS) containing proteinase K (200µg/mL) and DNA was subsequently isolated using phenol-chloroform and re-suspended in 50 μL of sterile water, as previously described.^16^

Generic HPV detection was performed by PCR with consensus primers MY09/11, for amplifying a 450 bp fragment of the HPV *L1* gene. The Supplementary data (Table I) shows all primers used in this study, including information on annealing temperature, genome position, amplicon sizes and references. Amplification was carried out in 50 μL reaction mixtures containing 1X PCR buffer, 200 μM dNTPs, 3 mM MgCl_2_, 50 pmol of each primer, 1.25 U of Platinum Taq DNA Polymerase and 5 μL of template. PCR conditions included a first step of 94ºC for 5 min, followed by 35 cycles of 94ºC for 30 s, 55ºC for 30 s and 72ºC for 1 min in a thermal cycler (Life Technologies, California, USA). PCR was completed following a final step of elongation at 72ºC for 10 min. A β-globin primer pair (10 pmol each), for amplifying a 330bp region of human DNA, was used as control. Positive controls of HPV16+ Ca Ski cell-line DNA and negative controls (water) were included in all reactions. PCR products were resolved on 1.5% agarose gels and stained with ethidium bromide. A 100-bp DNA ladder was run alongside the samples for size identification.

HPV16 identification was carried out by type-specific PCR with *E6* primers. Reaction mixtures, amplification conditions, and detection of products were the same as for generic HPV detection.

*Sodium bisulfite treatment and PCR amplification* - Bisulfite conversion and nested-methylation specific PCR (MSP) were performed according to previous reports.^17^ Briefly, approximately 1 µg of genomic DNA was denatured with NaOH (3 M) followed by bisulfite treatment at 50ºC for 16 h. Samples were subsequently purified with Wizard DNA Clean- Up System (Promega®, Madison, WI, USA), treated again with NaOH (3 M), precipitated with ethanol and resuspended in sterile water. For MSP, the first reaction was carried out with 5 µL of bisulfite-treated DNA and primers that did not discriminate between methylated and unmethylated nucleotides. Hence, amplicons were subjected to a second PCR step for identifying bisulfite-induced modifications of unmethylated cytosines, using two primer pairs (methylated - M, and unmethylated - U). Methylation of a specific gene was considered to be present if both, the specimen and a positive control, were amplified by M primers following treatment with sodium bisulfite. The Supplementary data (Figure) shows an agarose gel electrophoresis of unmethylated and methylated PCR amplified products. PCR findings were interpreted by two independent observers who had no access to cytology data.

*Disruption of E1 and E2 genes* - HPV integration into the host DNA genome frequently occurs with *E1* or *E2* disruption, resulting in suppression of *E2* transcription. Presence of a non-disrupted *E2* ORF was identified by PCR amplification of overlapping fragments, encompassing the *E1* and *E2* coding regions using 10 previously described primer pairs.^18^ PCR was carried out in 25 µL mixtures containing 0.2 mM of each dNTP, 25 pmol of each primer, 1 U of Platinum *Taq* DNA Polymerase (Life Technologies, California, USA) and 1 X buffer, 3 mM MgCl_2_. PCR conditions were: 95ºC for 5 min, followed by 35 cycles of 94ºC for 30 s, annealing temperature of 56ºC for 30 s, extension at 72ºC for 1 min, and final extension at 72ºC for 10 min. PCR products were visualised on 1.5% ultrapure agarose gels (Life Technologies, California, USA).

*Statistical analysis* - Data were analysed using GraphPad Prism 8.0 software. The extent of methylation at each CpG site per sample was estimated qualitatively as unmethylated or methylated cytosines. Statistical comparisons were performed using the chi-square test and Fisher’s exact test for categorical variables, between methylation levels and with and without *E1/E2* disruption in each group, based on cytological classification. A test for trend across ordered groups (*nptrend*) was used for verifying whether the number of samples with (i) a specific *E1/E2* integrity pattern (disrupted or intact) and (ii) with a *p16* ^*ink4a*^ promoter methylation pattern (unmethylated and methylated) followed a linear trend frequency in each group (LSIL, HSIL and CC).

**Fig. 2:**
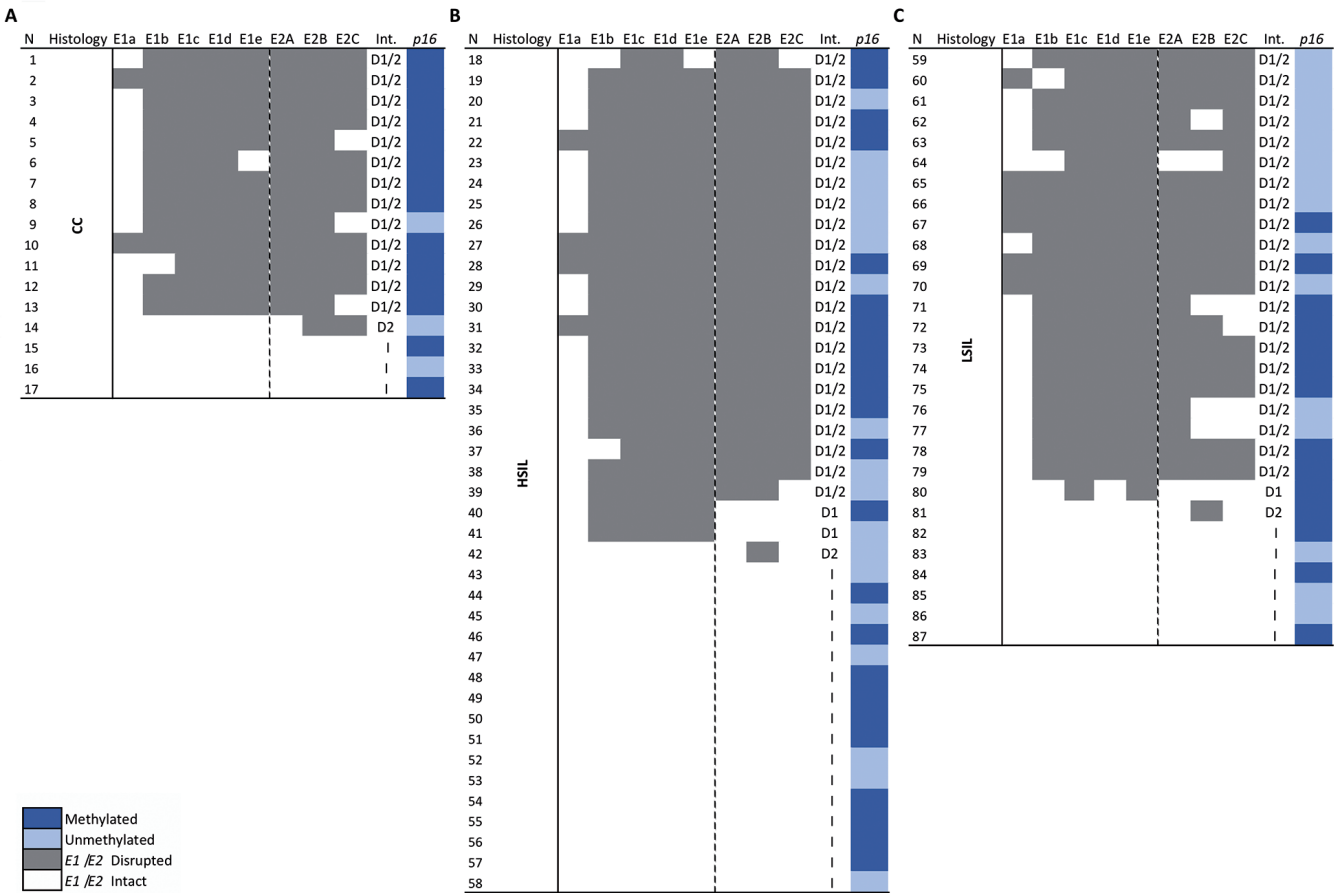
map of HPV16 *E1* and *E2* disruption sites and *p16* ^*ink4a*^ methylation per sample. (A) shows cervical cancer samples with predominant presence of *E1/E2* disruption (grey boxes) and methylated *p16* ^*ink4a*^ (dark blue boxes). (B and C) show the distribution of *E1/E2* disruption sites and *p16* ^*ink4a*^ in high-grade lesions (HSIL) and low-grade lesions (LSIL), respectively. Grey rectangles represent lack of polymerase chain reaction (PCR) amplification; white rectangles indicate presence of amplification. D1/2 indicates *E1* and *E2* disruption; D1: exclusive *E1* disruption; D2: exclusive *E2* disruption; I: intact *E1* and *E2*. E1a, E1b, E1c, E1d and E1e depict amplicons covering the *E1* gene. E2A, E2B and E2C, represent amplicons covering the *E2* gene. Int = integrity pattern; N = number of samples.

*Ethics* - This study was approved by Ethical Committee of Hospital Universitário Pedro Ernesto (Universidade Federal Fluminense, Rio de Janeiro, Brazil; protocol number 421/11).

## RESULTS

*Characteristics of the study cohort* - Eighty-seven HPV16+ patients enrolled in this study showed a mean age of 39 years [standard deviation (SD) ± 12] and a median of 37, ranging from 18 to 76 years. Patients with CC showed a higher median age (44, SD ± 12) with respect to patients with HSIL (37; SD ± 13) and LSIL (32; SD ± 11). Among CC patients, one presented with adenocarcinoma (1/17, or 6%) and 16 with squamous cell carcinomas (16/17, or 94%).

*Integrity of E1/E2 genes* - PCR amplification of the *E1/E2* region was performed for identifying HPV16 integrity, suggestive of the presence of episomal HPV capable of expressing E2. Intact *E1/E2* was found in 18% (3/17) of CC, 39% (16/41) of HSIL and 21% (6/29) of LSIL samples. Concomitant *E1* and *E2* disruption, resulting in lack of amplification of these contiguous genes, was the most frequent pattern observed in all cytology groups: CC (76% - 13/17), HSIL (54% - 22/41) and LSIL (73% - 21/29). In 57% (50/87) of all samples, this loss was larger than 1.9 Kb (between nt 1254 and 3189) (HPV16REF; K02718.1) (grey rectangles in the Fig. 2). Exclusive disruptions were less frequent: *E1* disruptions were observed in 5% of HSIL and 3% of LSIL samples, whereas *E2* disruptions were observed in 6% of CC, 2% of HSIL and 3% of LSIL samples. There was no significant association (*np*_*trend*_ = 0.881) between cytology status and *E1/E2* integrity (Table I).

*Methylation of the p16* ^*ink4a*^ *promoter* - Samples were qualitatively identified as methylated and unmethylated, according to the methylation status of the *p16* ^*ink4a*^ promoter. Methylation was found in 48% (14/29) of LSIL samples, with a statistically significant trend (*np trend* = 0.0320) to increasing in HSIL (56% - 23/41) and CC (79% - 14/17) (Table I). No significant association was observed between presence of methylated samples and *E1/E2* integrity in any cytology group (Table II). An additional table file [Supplementary data (Table II)] shows a compilation of all molecular and clinical information (including cytology diagnosis, age, *E1/E2* integrity and *p16* ^*ink4a*^ methylation status) of the samples included in this study.

## DISCUSSION

Infection by high-risk HPV types is a necessary requirement for developing precursor lesions and invasive cervical cancer, albeit insufficient, on its own, to cause this cancer. Several modulators, such as epigenetic modifications of the host cells, mainly DNA methylation, have been reported as likely complementary events for developing CC.^19^

In this study, a significantly increased trend (*np*_*trend*_ = 0.0320) was observed in the proportion of methylated *p16* ^*ink4a*^ promoter along with CC progression, from low-grade lesions to high-grade lesions to CC. Our findings suggest that methylation of the *p16* ^*ink4a*^ promoter might play an important role in the early stage of CC because approximately 50% of LSIL samples displayed a methylated pattern. This association was in agreement with other studies using either, similar or different methodologies of methylation analysis.^19^ ^,^ ^20^ ^,^ ^21^ Furthermore, increased methylation of the *p16* ^*ink4a*^ promoter has also been reported in CC samples with respect to the normal epithelium.^22^

**TABLE I:** Distribution of samples with respect to *E1/E2* integrity and *p16* ^*ink4a*^ methylation by cytology diagnosis

Cytology	*E1/E2* integrity			*p16* ^*ink4a*^ methylation		
	Intact	Disrupted		Unmethylated	Methylated	
CC	3 (18%)	14 (82%)	*np*_*trend*_ = 0.881	3 (21%)	14 (79%)	*np*_*trend*_ = 0.0320
HSIL	16 (39%)	25 (61%)		18 (44%)	23 (56%)	
LSIL	6 (21%)	23 (79%)		15 (52%)	14 (48%)	

Chi2 test np trend; CC: cervical cancer; HSIL: high-grade squamous intraepithelial lesion; LSIL: low-grade squamous intraepithelial.

**TABLE II:** Distribution of p16^ink4a^ methylated or unmethylated samples by *E1/E2* integrity in each group of cytology diagnosis

Cytology	*p16* ^*ink4a*^ methylation	*E1/E2* intact	*E1/E2* disrupted	*p-value* ^*^
CC	Unmethylated	1 (33%)	2 (67%)	0.4647
	Methylated	2 (14%)	12 (86%)	
HSIL	Unmethylated	6 (33%)	12 (67%)	0.5158
	Methylated	10 (43%)	13 (57%)	
LSIL	Unmethylated	3 (20%)	12 (80%)	0.9244
	Methylated	3 (21%)	11 (79%)	

*: chi2 test or Fisher Exact Test; CC: cervical cancer; HSIL: high-grade squamous intraepithelial lesion; LSIL: low-grade squamous intraepithelial.

Methylation of the *p16* ^*ink4a*^ promoter was not associated with an intact *E1/E2* region, normally present in an episomal HPV DNA. We hypothesised that, in these cases, cells carrying viral episomes would show lower expression of E6/E7 oncoproteins through HPV E2 regulation, while methylation of the promoter of the *p16* ^*ink4a*^ tumor suppressor gene would reduce its expression, contributing to cancer development, mainly in early events following infection.^7^ ^,^ ^22^ ^,^ ^23^

Contrary to our predictions, a higher frequency of methylated samples had disrupted *E1/E2* genes rather than intact *E1/E2* genes, although this finding was not statistically significant (Table II). The observed trend in the increase of *p16* ^*ink4a*^ methylation along with disease progression (Table I) was probably independent of *E1/E2* integrity in various stages of the disease (Table II). In agreement with our findings, Nuovo et al.,^21^ by methylation-specific PCR in situ hybridisation, also found that hypermethylation of the p16 gene could be an early event in a small number of LGSIL cells, and not associated with HPV genome disruption and integration. Moreover, it is likely that our sample selection, based on HPV-type specific PCR, may have introduced a bias by increasing the number of samples with higher viral load due to the presence of episomal HPV genomes, resulting in the low correlation with p16 promoter methylation.

Methylation of the *p16* ^*ink4a*^ promoter or even of a region downstream of its transcription start site, seems to be a complex event, probably induced by passive smoking or oral contraceptives.^19^ ^,^ ^22^ We postulate that it might also be induced by a feedback mechanism to reduce *p16* ^*ink4a*^ transcription in presence of high levels of *p16* ^*ink4a*^ expression in transformed, HR-HPV infected cells. A model, associating feedback epigenetic regulation with gene expression in adaptation and evolution, has also been proposed.^24^ Additionally, positive regulation of DNA methyltransferase, DNMT1, by HPV16 E6, through repression of p53 tumor suppressor protein, has also been reported.^25^ Increased DNMT1 levels, associated with recognition and CpG methylation of hemi-methylated DNA (DNA with only one strand methylated), has been observed along with the development of CC, from normal squamous epithelium to low-grade lesions, from low-grade to high-grade lesions, and from normal to invasive carcinomas.^26^ Furthermore, in head and neck cancers, HPV+ tumors may present different methylation patterns from host cell DNA, involving pathways like apoptosis, cell cycle and non-coding RNA.^27^ This strongly points to the influence of HPV oncogenes upon the epigenetic machinery.

On the other hand, lack of association between intact *E1/E2* and *p16* ^*ink4a*^ methylation might have resulted from the small number of samples with intact *E1/E2,* in all lesion types. We found a lower frequency (18% - 3/17) of intact *E1/E2* in HPV16+ cervical cancers as compared to other reports employing a similar methodology for integration analyses, which reported up to 70% of intact viral DNA.^28^ ^,^ ^29^ Our findings indicate that *E1/E2* disruption is a common and early event during HPV16 infection, encompassing all cervical disease types, in 79% of LSIL, 61% of HSIL and 82% of cancers. It is still controversial whether LSIL cases, presenting *E1/E2* disruption, might be at a higher risk for malignant transformation. It is likely that integration of the viral DNA in early stages of cervical disease may result in a persistent HPV infection and early dysregulation of *E6* and *E7* expression. HPV16 *E1/E2* disruptions in pre-neoplastic lesions have also been found using a similar methodology, although at a lower frequency (15% in LSIL and 37% in HSIL) as compared to the current study.^10^ Our findings reveal that, in all cervical disease grades, disruptions affected both genes, accounting for a large deletion of > 1.9 Kb. Other researchers have suggested that *E1* and *E2* disruptions might be associated with disease grades or HPV genotypes; exclusive *E1* disruption in HPV16+ LSIL and higher *E2* disruption in HPV18+ than in HPV16+ cervical tumors have also been reported.^28^ ^,^ ^29^

Differences between our findings and those reported in literature may be attributed to intrinsic characteristics of cohorts, the sensitivity of the integration assay or sample size. As previously discussed, the PCR based *E1/E2* integrity assay, while detecting intact *E1/E2,* cannot rule out the presence of integration of tandem arrays of multiple viral genomes or the concomitant presence of both integrated and episomal DNA conformations.^10^ ^,^ ^28^ Moreover, mechanisms still poorly understood, such as viral DNA degradation and elimination from cells, could affect the HPV genome integrity, especially in early stages of the disease, and may represent a limitation of the *E1/E2* integrity assay used in this study.^30^ Thus, our finding regarding the higher proportion of *E1/E2* disruption in LSIL, may represent a limitation of the approach based on PCR amplification of E1 and E2. Further experiments, by exploring alternative methodologies, such as in situ hybridisation (ISH), could better determine the HPV DNA integration pattern. However, it is important to keep in mind that ISH also exhibits limitations, such as interobserver variability and it is more suitable for tissues instead of cells.^31^ ^,^ ^32^

*In conclusion* - Our study showed an increased trend of methylation of the *p16* ^*ink4a*^ promoter from low-grade lesions to high-grade lesions, and from high-grade lesions to cervical cancer in HPV16+ patients. Additionally, high frequency of *E1/E2* disruptions in HPV16+ low-grade lesions suggested that viral DNA integration into the cell genome was an early event in cervical disease. Moreover, the methylation status was likely to be independent of HPV16 integrity.
