# Supplementary material for: Methylation of p16 ink4a promoter is independent of human papillomavirus DNA physical state: a comparison between cervical pre-neoplastic and neoplastic samples
Source: Mem Inst Oswaldo Cruz. 2018 Dec 17;114:e180456. doi: 10.1590/0074-02760180456 (PMC6319029; doi:10.1590/0074-02760180456)
Supplement: Supplementary file 1 [file 1678-8060-mioc-114-e180456-s.pdf]

TABLE I

Description of used primers during polymerase chain reaction (PCR). The table describes sets of primers in each PCR assay [human papillomavirus (HPV) DNA detection, HPV16 typing, and p16ink4a methylation - Nested-MSP], including the sequence of each primer, nucleotide position at the genome of reference, amplicon size and literature which designed was based

Assay			Primers (5'-3')	Nt position	Amplicon size (bp)	Reference
HPV DNA detection	MY11	forward:	GC**M**CAGGG**W**CATAA**Y**AATGG	*	450	Bauer et al.[33]
	MY09	reverse:	CGTCC**M**A**RR**GGA**W**ACTGATC	*		
HPV16 typing	E6 HPV16 $_{P1}$	forward:	CCCAGAAAGTTACCACAG	128 - 262	135	Moljin et al.[34] Nucleotide position based on HPV16REF (GenBank: K02718.1)
	E6 HPV16 $_{P2}$	reverse:	TACTATGCATAAATCCCG			
E1/E2 gene integrity	E1a	forward:	CCATGGCTGATCCTGCAG	863 - 1219	357	Vernon et al.[18]
		reverse:	TCTCCTTTTTGCAGCTCT			
	E1b	forward:	GACAGCGGGTATGGCAAT	1254 - 1663	410	
		reverse:	CATTCCCCATGAACATGC			
	E1c	forward:	AATAAATCAACGTGTTGCGATTGG	1548 - 2084	537	
		reverse:	GTTTATAATGTCTACACATTGTTG			
	E1d	forward:	GGATTGTGCAACAATGTG	2072 - 2527	456	
		reverse:	TGGAGGGCATTTTAGTTG			
	E1e	forward:	CAACTAAAATGCCCTCCA	2529 - 2845	317	
		reverse:	CGCATGTGTTTCCAATAG			
	E2A	forward:	CGAGGACAAGGAAAACGA	2738 - 3189	452	
		reverse:	CTTGACCCTCTACCACAG			
	E2B	forward:	GGTTTATATTATGTTCATGAAGG	3220 - 3599	380	
		reverse:	TATGGGTGTAGTGTTACTATTACA			
	E2C	forward:	GTAATAGTAACACTACACCCATA	3596 - 3853	258	
		reverse:	GGATGCAGTATCAAGATTTG			
Nested-MSP	p16-outer1	forward:	GAAGAAAGAGGAGGGGTTGG	21974658 - 21974930	273	Palmisano et al.[35]. Nucleotide position based on Chr9 GRCh37.p13
	p16-outer2	reverse:	CTACAAACCCTCTACCCACC			
	p16-M1	forward:	TTATTAGAGGGTGGGGCGGATCGC	21974757 - 21974906	150	
	p16-M2	reverse:	GACCCCGAACCGCGACCGTAA			
	p16-U1	forward:	TTATTAGAGGGTGGGGTGGATTGT	21974756 - 21974906	151	
	p16-U2	reverse:	CAACCCCAAACCACAACCATAA			

M = A+C; R = A+G; W = A+T; Y = C+T; Nested-MSP = nested methylated specific PCR; TA = annealing temperature; Nt position = nucleotide position; *: variable depending HPV genotype.

TABLE II

Compilation of all molecular and clinical information. The table depicts all information regarding samples herein studied, including cytology [low-grade squamous intraepithelial cells (LSIL), high grade squamous intraepithelial cells (HSIL), and cervical cancer (CC)], age, E1/E2 integrity and *p16*^{ink4a} promoter methylation status

Sample id	cytology	age	E1/E2_integrity	p16 Methylation
Sample 01	LSIL	45	Disrupted in E1/E2	unmethylated
Sample 02	LSIL	51	Disrupted in E1/E2	unmethylated
Sample 03	LSIL	32	Disrupted in E1/E2	unmethylated
Sample 04	LSIL	52	Disrupted in E1/E2	unmethylated
Sample 05	HSIL	47	Disrupted in E1/E2	methylated
Sample 06	LSIL	38	Disrupted in E1/E2	unmethylated
Sample 07	LSIL	23	Disrupted in E1/E2	unmethylated
Sample 08	HSIL	48	Disrupted in E2	unmethylated
Sample 09	LSIL	26	Disrupted in E1/E2	unmethylated
Sample 10	LSIL	50	Disrupted in E1/E2	unmethylated
Sample 11	LSIL	52	Disrupted in E1/E2	methylated
Sample 12	LSIL	39	Disrupted in E1/E2	unmethylated
Sample 13	HSIL	25	Disrupted in E1/E2	methylated
Sample 14	HSIL	25	Disrupted in E1/E2	unmethylated
Sample 15	HSIL	26	Disrupted in E1/E2	methylated
Sample 16	HSIL	25	Disrupted in E1/E2	methylated
Sample 17	HSIL	37	Disrupted in E1/E2	unmethylated
Sample 18	LSIL	24	Disrupted in E1/E2	methylated
Sample 19	HSIL	34	Disrupted in E1/E2	unmethylated
Sample 20	HSIL	33	Disrupted in E1/E2	unmethylated
Sample 21	HSIL	45	Intact	unmethylated
Sample 22	HSIL	28	Disrupted in E1/E2	unmethylated
Sample 23	HSIL	34	Intact	methylated
Sample 24	LSIL	48	Intact	methylated
Sample 25	LSIL	36	Disrupted in E1/E2	unmethylated
Sample 26	HSIL	43	Disrupted in E1	methylated
Sample 27	HSIL	25	Intact	unmethylated
Sample 28	HSIL	76	Intact	methylated
Sample 29	HSIL	64	Disrupted in E1/E2	unmethylated
Sample 30	HSIL	47	Disrupted in E1/E2	methylated
Sample 31	CC - SCC	56	Disrupted in E1/E2	methylated
Sample 32	CC - SCC	37	Intact	methylated
Sample 33	HSIL	31	Intact	unmethylated
Sample 34	CC - SCC	44	Disrupted in E1/E2	methylated
Sample 35	HSIL	44	Disrupted in E1/E2	unmethylated
Sample 36	LSIL	43	Intact	unmethylated
Sample 37	CC - SCC	62	Disrupted in E2	unmethylated
Sample 38	HSIL	30	Disrupted in E1/E2	methylated
Sample 39	LSIL	25	Intact	methylated
Sample 40	CC - SCC	36	Disrupted in E1/E2	methylated
Sample 41	HSIL	37	Disrupted in E1/E2	methylated
Sample 42	HSIL	58	Disrupted in E1/E2	methylated
Sample 43	CC - SCC	43	Disrupted in E1/E2	methylated
Sample 44	CC - SCC	25	Disrupted in E1/E2	methylated
Sample 45	LSIL	30	Intact	unmethylated
Sample 46	LSIL	18	Disrupted in E1/E2	methylated
Sample 47	HSIL	41	Intact	methylated
Sample 48	CC - SCC	48	Disrupted in E1/E2	methylated
Sample 49	HSIL	28	Intact	methylated

Sample id	cytology	age	E1/E2_integrity	p16 Methylation
Sample 50	HSIL	38	Disrupted in E1	unmethylated
Sample 51	CC - SCC	58	Disrupted in E1/E2	methylated
Sample 52	LSIL	26	Intact	unmethylated
Sample 53	CC - SCC	30	Disrupted in E1/E2	methylated
Sample 54	HSIL	52	Intact	methylated
Sample 55	LSIL	38	Disrupted in E1/E2	methylated
Sample 56	LSIL	23	Disrupted in E1/E2	methylated
Sample 57	LSIL	30	Disrupted in E1/E2	methylated
Sample 58	CC - SCC	44	Intact	unmethylated
Sample 59	HSIL	36	Intact	methylated
Sample 60	HSIL	51	Disrupted in E1/E2	methylated
Sample 61	HSIL	25	Intact	unmethylated
Sample 62	LSIL	27	Disrupted in E2	methylated
Sample 63	HSIL	68	Disrupted in E1/E2	methylated
Sample 64	HSIL	38	Intact	unmethylated
Sample 65	LSIL	44	Disrupted in E1/E2	methylated
Sample 66	CC - SCC	34	Disrupted in E1/E2	unmethylated
Sample 67	LSIL	21	Disrupted in E1/E2	unmethylated
Sample 68	CC - SCC	44	Intact	methylated
Sample 69	LSIL	28	Disrupted in E1/E2	unmethylated
Sample 70	LSIL	28	Intact	methylated
Sample 71	HSIL	34	Intact	methylated
Sample 72	LSIL	51	Disrupted in E1	methylated
Sample 73	CC - Adeno	59	Disrupted in E1/E2	methylated
Sample 74	HSIL	31	Intact	methylated
Sample 75	HSIL	51	Intact	methylated
Sample 76	HSIL	41	Intact	methylated
Sample 77	CC - SCC	34	Disrupted in E1/E2	methylated
Sample 78	HSIL	29	Disrupted in E1/E2	methylated
Sample 79	HSIL	29	Disrupted in E1/E2	unmethylated
Sample 80	LSIL	25	Disrupted in E1/E2	methylated
Sample 81	HSIL	25	Disrupted in E1/E2	methylated
Sample 82	LSIL	42	Disrupted in E1/E2	methylated
Sample 83	HSIL	50	Intact	unmethylated
Sample 84	HSIL	50	Disrupted in E1/E2	unmethylated
Sample 85	HSIL	25	Disrupted in E1/E2	unmethylated
Sample 86	CC - SCC	48	Disrupted in E1/E2	methylated
Sample 87	CC - SCC	51	Disrupted in E1/E2	methylated

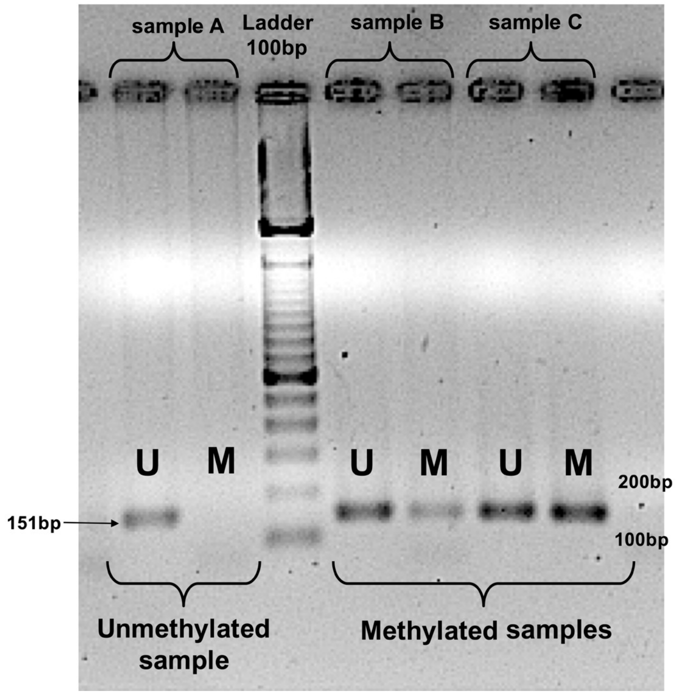

Products of cervical smear samples in the Nested-MSP. The gel (Agarose1.5%/TAE 1x) shows one unmethylated sample A (band positive on p16U and negative on p16M), and two methylated samples B/C positive for both primer pairs. Methylation of *p16*^{ink4a} gene was considered to be present if amplification was obtained by both, M and U primers, or only M positive, after sodium bisulfite modification. The molecular weight standard used was ladder 100bp (Invitrogen).
